# Refractory overactive bladder: a common problem?

**DOI:** 10.1007/s00192-015-2674-0

**Published:** 2015-03-20

**Authors:** Ulrich Schwantes, Joachim Grosse, Andreas Wiedemann

**Affiliations:** 1 Department of Medical Science/Clinical Research, Dr. R. Pfleger GmbH, 96045 Bamberg, Germany; 2 Urological Clinic, University Clinic Aachen, 52074 Aachen, Germany; 3 Department of Urology, Evangelisches Krankenhaus Witten gGmbH, University Witten/Herdecke, 58455 Witten, Germany

**Keywords:** Refractory OAB, Treatment, Antimuscarinic, Pathophysiology, Pharmacology

## Abstract

**Introduction and hypothesis:**

Unsatisfactory treatment outcome sometimes is described as frequently occurring in patients treated with first-line therapy for overactive bladder (OAB). The present article reviews the different circumstances which may result in failure to respond to lifestyle interventions, behavioral therapy, and/or antimuscarinic treatment.

**Methods:**

An extensive literature search was conducted to identify relevant articles on pathophysiological, clinical, and pharmacological aspects of refractory OAB.

**Results:**

Missing definition, unrealistic individual expectation of treatment outcomes, lack of communication between physician and patient as well as pathophysiological and pharmacological processes were identified as relevant for failure to respond to first-line OAB treatment. Increase of patient’s motivation to adhere to the prescribed treatment, critical examination of the patient in regard to the initial diagnosis, and individual adjustment of antimuscarinic therapy may be appropriate tools to improve treatment outcome in OAB patients.

**Conclusions:**

Overall, the incidence of refractory OAB seems to be overestimated. There are several approaches to improve therapy results.

## Introduction

Lifestyle interventions and behavioral therapies, which may be combined with antimuscarinic treatment, are recommended in international guidelines as first-line therapy for patients with overactive bladder (OAB) [1, 2]. Failure to respond to conservative and pharmacological treatment of OAB has previously been described [3], but there is considerable heterogeneity in the definitions of both treatment response and nonresponse in trials involving patients with OAB [4]. Over the last few years several clinical studies have been published which investigated different pharmacological principles (e.g., other antimuscarinic drugs, beta-3 agonists, botulinum toxin) and strategies (increasing anticholinergic doses, additional treatment with other anticholinergic drugs, or beta-3 agonists) in the treatment of patients with refractory OAB [5–10]. The vast majority of these studies report significant improvement of OAB symptoms following a change in treatment. In this review we discuss published data concerning factors which may be behind refractory OAB in order to aid in understanding different treatment results in OAB patients.

## Materials and methods

Computerized library systems such as MEDLINE, BIOSIS, and EMBASE were analyzed regarding articles on refractory OAB published between 2000 and 2014. Search terms included “overactive bladder” or “detrusor overactivity” or “antimuscarinic” AND “non-responder,” or “refractory,” or “fail,” or “persistent,” or “dissatisfied.” Furthermore, reviews of pharmacological and physiological aspects were carried out using the same first two search terms combined with terms such as “muscarinic,” “nicotinic,” “m-receptor,” “adrenergic,” and “transporters.” In addition, unpublished pharmacological and clinical data were included.

## Results

### The missing definition

Refractory OAB patients most likely represent a minority of the total OAB population, but the epidemiology is unknown [11]. As shown by Goldman et al., a wide variety of symptom-based definitions and patient-reported outcomes with inconsistent thresholds are used in the published literature to decide whether or not patients respond to conservative and/or antimuscarinic treatment [4]. Moreover, patients’ individual evaluation of treatment success is characterized by different expectations and perceptions [4]. Accordingly, patients’ statements on failure of antimuscarinic treatment are diverse. A large-scale study involving 5,392 patients showed that 46.2 % of those who reported discontinuing one or more antimuscarinic OAB medications gave the reason for this that the treatment “did not work as expected,” in 25.1 % medication was switched, 23.3 % “learned to get by without medication,” and 21.1 % discontinued due to side effects [12]. The current American Urological Association (AUA) and European Association of Urology (EAU) guidelines on urinary incontinence contain recommendations for the treatment of refractory OAB [1, 2], but no definitions of criteria and/or thresholds to assess unsatisfactory outcomes of therapy were described.

### Physician’s expectations

Current clinical studies in patients with refractory OAB whose behavioral and/or antimuscarinic treatment was stopped due to lack of efficacy or side effects and replaced by alternative medication (e.g., botulinum toxin A, other antimuscarinics, beta-3 agonists) are summarized in Table 1. When assessing the results of these studies concurrently, it becomes clear that definitions of “antimuscarinic failure” are based on inconsistent criteria ranging from the somewhat subjective evaluation “lack of benefit or intolerable side effects” to the strict prerequisite of “≥1 UUI/day.” This corresponds with the observations made by Goldman et al. in their systematic review [4]. If the criteria for diagnosis of refractory OAB used in these studies are compared with those defined for success in the changed treatment studies (Table 1), it becomes obvious that the latter criteria often seem to be less strict [6, 13–15]. If the same strict criteria of > 1 UUI day, as was chosen for inclusion into the study by Kanagarajah et al. [15] and Kuo [13], had been defined as the response following secondary therapy, practically no patients could be classified as a responder in the described clinical studies with botulinum toxin A. This demonstrates that the physician’s individual expectations may be highly relevant in differentiating between responders and nonresponders to primary OAB treatment. In practice, complete cure of OAB symptoms under first-line treatment is rare [16], and even after second-line treatment with botulinum toxin and neuromodulation the proportion of patients showing improvement of symptoms rather than total cure is greater [17, 18].

**Table 1:** Clinical studies in patients with refractory OAB investigating efficacy of changed pharmacological therapy

Study design	Pretreatment	Diagnosis of refractory OAB	Treatment/sample size	Criteria for treatment success	Study results after treatment (change from baseline)	Reference
Single-blind, actively controlled	Behavioral modification + antimuscarinics	Urodynamic overactive detrusor, plus at least 1 episode of urgency/day or 1 UUI/day	OnabotulinumtoxinA/105	>2 scale changes in PPBC	IS: UUI episodes: BB: 7.07 (−9.23) BB/trigone: 5.28 (−7.62) BBase/trigone 15.8 (−6.24)	Kuo [13]
Double-blind, placebo-controlled	Antimuscarinic	As per investigator opinion: Qmax > 12 ml/s IPSS > 12 IPSS QOL > 3	OnabotulinumtoxinA/28 (15 vs 13 placebo)	Reduction in urinary frequency	Frequency (day 90): Placebo: 13 (+2.5) Botulinum toxin: 8 (−3)	Chughtai et al. [14]
Open uncontrolled	Antimuscarinic	1 UUI/ day and/or >9 voids/day	Botulinum toxin A/32	>50 % reduction in urinary frequency/day	UUI episodes: (−50 %)	Kanagarajah et al. [15]
Open uncontrolled	Antimuscarinic	Lack of benefit or intolerable side effects	Solifenacin/9	Daytime frequency, nocturia, micturition volume, and UUI/day	UUI episodes: 1.9 (−3.0) Nocturia/day: 0.9 (−1.9) Daytime micturitions/day: 7.3 (−4.1)	Wong & Duggan [9]
Open cohort study	Antimuscarinic	No improvement of symptoms (not specified)	Mirabegron alone or in combination with antimuscarinic	PGI-I	PGI-I: Very much better: 9 % Much better: 25 %	Balachandran et al. [6]
Double-blind, placebo-controlled	Antimuscarinic	Suboptimal response (≤50 % reduction in UUI episodes during 2 weeks run-in)	Fesoterodine/536	Decrease of UUI episodes	UUI episodes Fesoterodine: (−2.37) Placebo: (−1.87)	Kaplan et al. [10]

*PPBC* patient’s perception of bladder condition, *IS* injection site, *UUI* urge urinary incontinence episodes, *BB* bladder body, *IPSS* International Prostate Symptom Score, *QOL* quality of life, *PGI-I* Patient Global Impression of Improvement

### Patients’ motivation and expectations

Essential for treatment response to OAB treatment is adherence to the prescribed therapy. Although it seems mundane, during both conservative and antimuscarinic treatment, realistic information provided to the patient on possible treatment efficacy, and on side effects, and additionally the quality of the course for lifestyle modification and behavioral bladder retraining, are essential to reach adherence to the prescribed treatment [1, 16]. Lifestyle modification should include stopping smoking and limiting the intake of caffeine, alcohol, and carbonated and citrus beverages, which may lead to an increase in OAB symptoms [16, 19].

Indeed, inadequate follow-up after initiation of therapy (poor motivation) and unmet or unrealistic expectations (poor communication between patients and the physician) have been identified as contributory factors to nonadherence, in addition to adverse events and insufficient beneficial effects [20]. Jundt et al. demonstrated in their survey that 10 % of patients with OAB had not started with the medication 12 months after prescription because of their “fear of side effects” or did “not want to take pills” [21]. In this study, most patients stopped taking the medication without discussing the issue with their doctor. Swartz and Vasavada assume that many patients may be prematurely labeled as having refractory OAB after only a modest attempt at medical or behavioral treatment [11]. Consequently, regular follow-ups to monitor treatment effects and adherence may be useful [16] as well as providing realistic information on symptom improvements as expected treatment success and on any side effects which may occur.

### Pathophysiological reasons

OAB is a symptom complex rather than a disease. Lack of efficacy of treatment with antimuscarinics as well as with behavioral bladder retraining, may be associated with underlying causes for lower urinary tract dysfunction which may have been overlooked in previous examinations. In order to exclude any other causes, repeated examinations of bacterial cystitis, painful bladder, voiding dysfunction, bladder or pelvic tumors, calculus, atrophic vaginitis, vaginal prolapse, medication side effects, neurological reasons, polyuria (polydipsia, diabetes mellitus/insipidus, chronic renal failure, and hyperthyroidism) should be considered [1, 22, 23].

Digesu et al. investigated 110 women with refractory OAB by cystoscopy and biopsy [24]. The patients had undergone conservative management (lifestyle change, bladder retraining, and physiotherapy) and previous treatment with two or more antimuscarinics. Among the patients, histopathology showed chronic cystitis in 94, follicular cystitis in 3, acute and chronic cystitis in 2, transitional cell carcinoma in 6, and no abnormality in 1, suggesting that OAB refractory to antimuscarinics may be caused by chronic inflammation.

Evidence that inflammatory processes are involved in the pathogenesis of refractory OAB emerged from the investigations by the research group of Kuo who found that the inflammatory markers serum nerve growth factor (NGF), C-reactive protein, and adipokines including interleukins and tumor necrosis factor are increased in patients with refractory OAB [13, 25, 26]. Interestingly, elevated urinary NGF levels decreased significantly in 39 women with refractory OAB after antibiotic therapy, while the OAB symptoms daytime frequency, nocturia, and urgency simultaneously improved significantly [27]. Seventy four percent of these women also reported improvement in perception of their bladder condition.

### Pharmacological reasons

#### Pharmacokinetics

After oral administration, bioavailability of the tertiary antimuscarinic drugs darifenacin, oxybutynin, fesoterodine, and tolterodine as well as of the quaternary drug trospium chloride is characterized by a high intersubject variability (Table 2) ([28–30], data on file 2013, Dr. R. Pfleger GmbH, Bamberg, Germany). In the case of the tertiary antimuscarinics, individual pharmacokinetics may vary due to genetic differences resulting in dissimilar metabolic degradation by enzymes of the cytochrome P450 system [29, 31]. In contrast, variability of bioavailability of trospium chloride is primarily based on the relatively low rate of intestinal absorption [32], and excretion via bile and urine, where different drug carriers seem to be involved [33, 34].

**Table 2:** Intersubject variability of maximum plasma concentration (C_max_) and area under the curve (AUC) values of different antimuscarinic drugs under steady-state conditions

		Reference
Variability of C_max_ in %		
Fesoterodine ER	33–48	Malhotra et al. [28]
Tolterodine ER	46–87	Malhotra et al. [28]
Trospium chloride IR	9–64	Data on file 2013, Dr. R. Pfleger GmbH, Bamberg
Variability of AUC in %		
Darifenacin ER	48–71 % (EM)	Skerjanec [29]
	20–61 % (PM)	

*ER* extended release, *IR* immediate release, *PM* poor metabolizers, *EM* extensive metabolizers

Furthermore, the pharmacokinetics of some antimuscarinic drugs are influenced by food intake. Increased C_max_ values of the active drug were observed when oxybutynin, darifenacin, and fesoterodine extended release were coadministered with a high-fat meal [29, 31, 35], whereas decreased C_max_ and AUC values were calculated after ingestion of a high-fat meal together with trospium chloride [36].

Further factors which can influence the pharmacokinetics of antimuscarinics include age, gender, and race (Table 3) as well as hepatic and renal impairment. Since drug excretion via the kidneys declines with age, Turnheim recommends treating the elderly in general as renally insufficient patients [45].

**Table 3:** Influences of age, gender, and race on pharmacokinetics of different antimuscarinics used for OAB

Factor influencing PK	Antimuscarinic drug	Influence	Reference
Age	Darifenacin	Yes	Skerjanec [29]
	Fesoterodine	No	Malhotra et al. [37]
	Oxybutynin	Increased plasma levels	Hughes et al. [38]
	Solifenacin	AUC + 20 %	Krauwinkel et al. [39]
	Tolterodine	No	Wefer et al. [40]
	Trospium chloride	No	Doroshyenko et al. [41]
Gender	Darifenacin	Lower CL in women	Kerbusch et al. [42]
	Fesoterodine	No	Malhotra et al. [37]
	Oxybutynin	No	Lukkari et al. [43]
	Solifenacin	No	Krauwinkel et al. [39]
	Tolterodine	No	Guay [44]
	Trospium chloride	No	Data on file 2013, Dr R. Pfleger GmbH
Race	Darifenacin	Japanese: lower BA	Skerjanec [29]
	Fesoterodine	Japanese	Malhotra et al. [37]
	Tolterodine	Whites: AUC + 10 %	Guay [44]

*PK* pharmacokinetics, *BA* bioavailability, *CL* clearance

#### Interactions

In addition to the described variations of the pharmacokinetics of antimuscarinics, interactions with other simultaneously taken drugs may lead to decreased or increased effects, thereby possibly limiting treatment efficacy or tolerability. It should be taken into account that pharmacokinetic interactions may occur by competition in absorption, metabolic processes, and excretion. Pharmacodynamic interactions may be caused in particular by concomitant medication which can also have additional anticholinergic potency. As shown by Sumukadas et al., the proportion of older people with a very high anticholinergic exposure increased from 7.3 % in 1995 to 9.9 % in 2010 [46]. It is known that a greater anticholinergic burden can lead to significant deficits in cognitive function [47].

#### Muscarinic receptors

Pharmacodynamic reasons for refractory OAB could also be based on the receptor level. Age-related decrease of muscarinic receptors in the urinary bladder has been shown in rats, but there were only minor, if any, alterations in receptor responsiveness [48]. In humans, a shift of M_3_ muscarinic receptor subtypes to M_2_ was observed in correlation with age and in patients with neurogenic bladder overactivity [32]. Whether such changes are of clinical relevance still remains to be clarified.

All the described factors may necessitate adjustment of individual doses in antimuscarinic treatment. A decrease of the daily dose may be an option especially in elderly patients when side effects become problematic, as physiological changes connected with aging may result in reduced metabolism, alterations in distribution, declined excretion, and a decline in counter-regulatory mechanisms [45].

### Pharmacological management of patients not responding to first-line therapy

Generally, flexible dosing with antimuscarinics provides the opportunity to increase or decrease the dose to meet the body habitus and the pharmacokinetic and clinical needs of the individual patients, thereby balancing efficacy against tolerability [49]. In connection with this, discussion and exchange of information between the physician and patient are a definite requirement to define the individual dose.

**Fig. 1:**
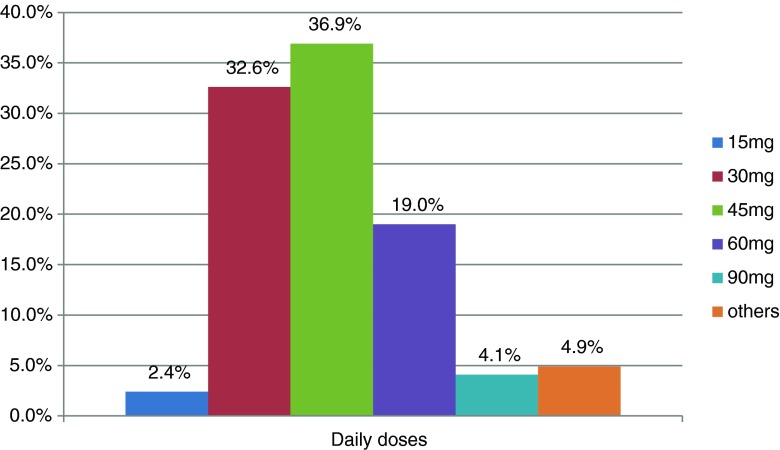
Different daily doses of trospium chloride immediate release tablets prescribed by German urologists in 9,366 patients with OAB symptoms in medical practices. Pooled data from three non-interventional studies carried out in Germany (data on file, 2014, Dr. R. Pfleger GmbH, Bamberg, Germany)

Data collected from non-interventional studies show that flexible dosing of trospium chloride adjusted to the patient’s individual needs is commonly used in urological practices in Germany (Fig. 1) (data on file, 2014 Dr. R. Pfleger GmbH, Bamberg, Germany).

In patients who failed to respond, or showed suboptimal response to antimuscarinic drugs but tolerated the treatment well, it was shown that an increase of the daily dose may lead to a significant improvement of the OAB symptoms. A significant decrease of incontinence episodes was observed after doubling the recommended daily doses of trospium chloride and tolterodine to 90 and 8 mg, respectively, in patients with persistent neurogenic detrusor overactivity (NDO) [8]. An alternative approach is the combination of two different antimuscarinic drugs, which has been demonstrated as effective and safe in several clinical studies including patients with either OAB or NDO [7, 50–53]. Combination of an antimuscarinic with a beta-3 agonist has previously been investigated in clinical studies (Table 4) [6, 54].

**Table 4:** Clinical studies using increased [8] or combined antimuscarinic treatment in patients with unsatisfactory benefit of previous/initial therapy

Medication at start of therapy	Adjustment and dose/day	Patients (*n*)	Efficacy results	Reference
Tolterodine 1 × 4 mg/day (*n* = 11) Trospium 3 × 15 mg/day (*n* = 10)	Tolterodine 2 × 4 mg/day (*n* = 11) Trospium 3 × 30 mg/day (*n* = 10)	NDO (21)	In total UI episodes decreased from 8–12 to 0–2	Horstmann et al. [8]
Oxybutynin 30 mg/day Tolterodine 2 × 8 mg/day Trospium 3 × 30 mg	Plus trospium 45–90 mg Plus oxybutynin 15–30 mg Plus tolterodine 4–8 mg	NDO (27)	UI episodes decreased: From 8.6 ± 2.7 to 1.3 ± 0.9 From 7.0 ± 1.5 to 0.6 ± 0.7 From 7.5 ± 2.7 to 2.0 ± 1.5	Amend et al. [50]
Tolterodine 4 mg	Plus solifenacin 5 mg Plus solifenacin 10 mg	NDO (19)/OAB (14)	UI episodes decreased: By 100 % in 17 patients By > 90 % in 14 patients By 50–89 % in 2 patients	Bolduc et al. [53]
Oxybutynin 15 mg/day Oxybutynin 15 mg/day	Plus trospium 80 mg Plus solifenacin 10 mg	NDO (12)	Decrease of UI episodes: From 5.3 ± SD to 0.8 ± SD From 4.5 ± SD to 1.0 ± SD	Nardulli et al. [52]
Oxybutynin or Tolterodine	Plus tolterodine ER 4 mg or Plus solifenacin 5 mg or 10 mg	NDO/OAB (31/25)	Decrease of UI episodes: By 100 % in 23 patients By > 90 % in 18 patients By 50–89 % in 15 patients	Nadeau et al. [7]
Trospium 60 mg plus solifenacin 20 mg (198) or Placebo (115)		OAB (313)	Significant decrease in UI episodes compared to placebo	Kosilov et al. [51]
Solifenacin 2.5, 5, or 10 mg or Mirabegron 25 or 50 mg or Solifenacin + mirabegron, or Placebo		OAB (1306)	Dose–response relationship for MVV in all combination groups No significant changes in UI episodes compared to placebo	Abrams et al. [54]

*UI* urinary incontinence, *NDO* neurogenic detrusor overactivity, *OAB* overactive bladder (number of patients in parentheses), *ER* extended release, *MVV* volume voided per micturition

When assessing these study results in patients with refractory OAB, after doubling the recommended dose, or after combination treatment with a second drug, or after replacement by another antimuscarinic, one should consider that the situation of the unsatisfactory response to behavioral and antimuscarinic therapy is observed during daily routine medical practice. Patients with OAB who are included in clinical trials receive more intensive monitoring of their treatment. Motivation of patients with OAB may be increased by a defined regular follow-up appointment during clinical studies, and specified selection criteria could lead to a relatively homogeneous patient population. Consequently, symptom improvement in such clinical trials may not be reproducible during clinical practice, when antimuscarinic treatment is not accompanied by close patient guidance [6]. This also applies to trials involving patients with refractory OAB, where treatment with an antimuscarinic drug was replaced by another antimuscarinic or a beta-3 agonist, such as mirabegron [6, 9].

However, dose adjustment, the combination of two different antimuscarinic drugs, and replacement by another antimuscarinic or a beta-3 agonist are options in order to improve treatment success in individual patients with OAB. Selection of the best tolerated drug is of great importance to avoid unnecessary side effects. Especially in older patients where co-medication can have its own anticholinergic effects and/or compete in metabolism in the cytochrome P450 system, impairment of cognitive function as a side effect, and metabolic interactions, can largely be avoided by choosing an appropriate antimuscarinic such as the quaternary molecule trospium chloride, which does not seem to contribute to such effects [55–58] and enables flexible dosing.

In patients in whom critical reassessment of diagnosis leads to an exclusion of underlying causes for lower urinary tract dysfunction and who do not respond to intensive first-line therapy including adjusted antimuscarinic treatment, the use of transcutaneous electrical nerve stimulation, the intravesical administration of onabotulinumtoxinA injections, or augmentation cystoplasty may be further treatment options [59].

## Conclusion

The incidence of refractory OAB seems to be overestimated. An unsatisfactory improvement of symptoms in the first-line treatment option of patients with OAB may depend on various factors. Realistic estimation of treatment outcomes and side effects by the patient and/or the physician, individual patient guidance to improve the patient’s motivation to adhere to treatment, review of the OAB diagnosis, and exclusion of other underlying causes as well as individual antimuscarinic dose adjustment are procedures which can improve the success of OAB therapy in daily medical practice. The authors suggest using the term refractory OAB only in such cases when the aforementioned steps have confirmed the patient’s nonresponse to first-line treatment including antimuscarinic therapy.
